# Synaptic expression of TAR-DNA-binding protein 43 in the mouse spinal cord determined using super-resolution microscopy

**DOI:** 10.3389/fnmol.2023.1027898

**Published:** 2023-08-21

**Authors:** Matthew J. Broadhead, Ani Ayvazian-Hancock, Katherine Doucet, Owen Kantelberg, Lesley Motherwell, Fei Zhu, Seth G. N. Grant, Mathew H. Horrocks, Gareth B. Miles

**Affiliations:** ^1^School of Psychology and Neuroscience, University of St. Andrews, St. Andrews, United Kingdom; ^2^Centre of Biophotonics, University of St. Andrews, St. Andrews, United Kingdom; ^3^Edinburgh Super-Resolution Imaging Consortium, Heriot-Watt University, Edinburgh, United Kingdom; ^4^EaStCHEM School of Chemistry, University of Edinburgh, Edinburgh, United Kingdom; ^5^Genes to Cognition Program, Centre for Clinical Brain Sciences, University of Edinburgh, Edinburgh, United Kingdom; ^6^Simons Initiative for the Developing Brain (SIDB), Centre for Discovery Brain Sciences, University of Edinburgh, Edinburgh, United Kingdom; ^7^IRR Chemistry Hub, Institute for Regeneration and Repair, University of Edinburgh, Edinburgh, United Kingdom

**Keywords:** ALS (Amyotrophic Lateral Sclerosis), synapse, super-resolution, TDP-43 (43 kDa TAR DNA-binding protein), spinal cord

## Abstract

Amyotrophic Lateral Sclerosis (ALS) is characterised by a loss of motor neurons in the brain and spinal cord that is preceded by early-stage changes in synapses that may be associated with TAR-DNA-Binding Protein 43 (TDP-43) pathology. Cellular inclusions of hyperphosphorylated TDP-43 (pTDP-43) are a key hallmark of neurodegenerative diseases such ALS. However, there has been little characterisation of the synaptic expression of TDP-43 inside subpopulations of spinal cord synapses. This study utilises a range of high-resolution and super-resolution microscopy techniques with immunolabelling, as well as an aptamer-based TDP-43 labelling strategy visualised with single-molecule localisation microscopy, to characterise and quantify the presence of pTDP-43 in populations of excitatory synapses near where motor neurons reside in the lateral ventral horn of the mouse lumbar spinal cord. We observe that TDP-43 is expressed in approximately half of spinal cord synapses as nanoscale clusters. Synaptic TDP-43 clusters are found most abundantly at synapses associated with VGLUT1-positive presynaptic terminals, compared to VGLUT2-associated synapses. Our nanoscopy techniques showed no difference in the subsynaptic expression of pTDP-43 in the ALS mouse model, SOD1^G93a^, compared to healthy controls, despite prominent structural deficits in VGLUT1-associated synapses in SOD1^G93a^ mice. This research characterises the basic synaptic expression of TDP-43 with nanoscale precision and provides a framework with which to investigate the potential relationship between TDP-43 pathology and synaptic pathology in neurodegenerative diseases.

## Introduction

Amyotrophic Lateral Sclerosis (ALS) is characterised by a progressive loss of motor control due to a loss of motor neurons (MNs) in the spinal cord, brain stem, and motor cortex. Prior to MN loss, structural and functional changes in synapses have been reported between neurons in both the brain and spinal cord (Fogarty et al., 2015, 2016; Jiang et al., 2019; Bączyk et al., 2020; Broadhead et al., 2022). Synaptopathy in ALS is characterised by early stage hyper-excitability, that may induce excitotoxicity, and a later stage loss of vulnerable synapse subtypes (Van Zundert et al., 2008; Fogarty, 2019; Broadhead et al., 2022). The molecular mechanisms of ALS synaptopathy, however, are not fully understood.

TAR-DNA-Binding Protein 43 (TDP-43) is a ubiquitously expressed DNA/RNA binding protein that provides critical roles in RNA splicing, translation and transport in the nucleus and cytoplasm of neurons and glia in the nervous system (Ayala et al., 2008, 2011; Tollervey et al., 2011). Mutations in the gene encoding TDP-43 (TARDBP) have been identified in 4–5% of familial ALS cases and 1% of sporadic ALS cases (Sreedharan et al., 2008; Millecamps et al., 2010). The aberrant accumulation of misfolded, hyper-phosphorylated TDP-43 (pTDP-43) in the cytoplasm of neuronal and glial cells also occurs in over 90% of all ALS cases (Mackenzie et al., 2010).

TDP-43 may play a role at synapses in both healthy and diseased conditions (Ling, 2018). TDP-43-bound mRNAs encode proteins that have roles in presynaptic and postsynaptic function, such as the glutamate receptor GluA1, glial excitatory amino acid transporter-2 (EAAT2) and microtubule-associated protein Map1b (Godena et al., 2011; Sephton et al., 2011; Tollervey et al., 2011; Colombrita et al., 2012). The expression of TDP-43 has been observed in the dendrites of cultured rat hippocampal neurons and colocalised with markers of the postsynaptic density (PSD) of excitatory synapses (Wang et al., 2008). Genetic alteration of the expression levels of TDP-43 in ALS mouse models is associated with both increased and decreased dendritic branching and synapse number, suggesting a role in regulating the structural plasticity of synapses (Majumder et al., 2012; Fogarty et al., 2016; Ling, 2018; Jiang et al., 2019). Analyses from human patients with ALS and the related cognitive disorder, frontotemporal dementia (FTD), suggest a strong correlation between TDP-43 pathology, synapse loss and the severity of cognitive symptoms (Henstridge et al., 2018).

Taken together, synaptic dysfunction may be linked to TDP-43 pathology. However, there has been little characterisation of the synaptic expression of TDP-43 in spinal cord synapses. MNs, for example, receive numerous different types of synaptic inputs, from excitatory, inhibitory and modulatory synapses derived from descending inputs from the brain, local spinal cord interneurons and sensory neurons. Not all synapses are equal, as some may be more selectively targeted in certain disease conditions (Mentis et al., 2011; Sunico et al., 2011; Herron and Miles, 2012; Bączyk et al., 2020; Broadhead et al., 2022). Understanding the expression of TDP-43 in different types of synapses in the spinal cord may provide insights into the selective vulnerability of certain synapses and pathways in ALS and therefore advance knowledge of the pathogenic mechanisms underlying this devastating disease.

In this study, we have used high-resolution and super-resolution microscopy techniques to visualise and assess the presence of phosphorylated TDP-43 (pTDP-43) at synapses within the mouse spinal cord.

## Methods

### Animals and ethics

All procedures performed on animals were conducted in accordance with the UK Animals (Scientific Procedures) Act 1986 and were approved by the University of St Andrews Animal Welfare and Ethics Committee. The B6SJL-TgN(SOD1-G93A)1Gur/J (SOD1) mouse line was kindly provided by Dr. Richard Mead. PSD95-eGFP mice were originally obtained from Prof. Seth Grant (University of Edinburgh). The PSD95-eGFP mouse is a genetically engineered in-frame fusion knock-in mouse, bred on a C57BL/6J background, using a strategy detailed previously (Fernández et al., 2009; Broadhead et al., 2016; Zhu et al., 2018). The enhanced green fluorescent protein (eGFP) is fused to the C-terminal of the endogenous PSD95 protein, enabling the visualisation of postsynaptic densities (PSDs) of excitatory synapses throughout the nervous system (Broadhead et al., 2016; Zhu et al., 2018; Cizeron et al., 2020). As characterised previously, SOD1 x PSD95-eGFP progeny display similar weights and behavioural phenotypes as expected from ‘pure’ SOD1 mice, with the onset of hind-limb tremors and reduced hind-limb splay by approximately 75 days of age (Mead et al., 2011; Broadhead et al., 2022). Mice were all aged 111 days old at the point of sacrifice and tissue collection. A total of 11 mice were used in this study, as a result of breeding a SOD1 male with homozygous PSD95-eGFP females. The resultant progeny of 11 mice were all heterozygous for PSD95-eGFP; whilst 5 of the mice did not have the mutant SOD1 gene (healthy control litter mates) and 6 of the mice did express the mutant SOD1 gene.

### Spinal cord tissue collection

Mice were anaesthetised with pentobarbitol (30 mg/kg dose, Dolethal), and the chest cavity opened to reveal the heart. The right atrium was severed and 10 mL ice cold 1 × phosphate buffered saline (PBS) was perfused through the left ventricle, followed by 10 mL 4% paraformaldehyde (PFA; Alfa Aesar). The lumbar spinal cord was then dissected and incubated for a further 3–4 h (h) in 4% PFA before being incubated in sucrose 30% w/v for up to 72 h at 4°C until sunk. Prior to cryo-embedding, the spinal cord was cut at approximately Lumbar segment 1 (L1), which was determined as being approximately 2 mm rostral of the lumbar enlargement, and by examining the distribution of the ventral roots. Thus, the first cryosections were taken at approximately Lumbar segment 1–2 (L1-2). Tissue was then cryo-embedded in OCT compound and stored at − 80°C. Cryosections at 20 μm thickness were obtained using a Leica CM1860 cryostat and adhered to Superfrost Gold Plus glass slides (VWR). For experiments requiring total internal reflection fluorescence (TIRF) microscopy, 10 μm thick sections were adhered to an Argon Plasma cleaned coverslip (1.0 thickness).

### Immunohistochemistry and aptamer labelling

For immunohistochemistry, slides with spinal cord slices were first heated at 37°C for 30 min to aid the adherence of the tissue to the glass slides and reduce tissue loss during subsequent wash steps. Slides were washed three times in PBS before being blocked and permeabilised in PBS containing 3% Bovine Serum Albumin (BSA), 1% donkey serum and 0.2% Triton X100 for 2 h at room temperature. Primary antibodies were diluted 1:500 in PBS containing 1.5% BSA and 0.1% Triton X100, and samples were incubated with primary antibody solution for 2 nights at 4°C. Primary antibodies used include: Anti-SMI32 (Mouse, Biolegend, 801701) Anti-VGLUT2 (Mouse, Abcam, ab79157), anti-VGLUT1 (Guinea Pig, Milipore, AB5905), and two different anti-pTDP-43 antibodies (Rabbit, Cosmo Bio, CAC-TIP-PTD-P03; Rabbit, ProteinTech, 22309-01). Once incubated, slides were washed five times in PBS over the course of 1–2 h. Secondary antibodies were diluted 1 in 500 in PBS with 0.1% Triton X100, and tissue was incubated in secondary antibody solution for 1.5–2 h at room temperature followed by a further five washes in PBS over the course of 1–2 h. Secondary antibodies used were Donkey-anti-Rabbit conjugated to Alexa Fluor 555 (Abcam, ab150062), Donkey-anti-Mouse conjugated to Alexa Fluor 647 (Abcam, ab150107), Donkey anti-Mouse conjugated to Alexa Fluor 405 plus (Invitrogen, A48257) and Donkey anti-Guinea Pig conjugated to Alexa Fluor 647 (Jackson Laboratories, 706-605-148). Finally, slides were dried and coverslips of 0.17 mm thickness were adhered using hard-setting Prolong^™^ Glass Antifade Mountant (Invitrogen).

### TDP-43 aptamer generation and labelling

The generation of the TDP-43 RNA aptamer is described previously by Zacco et al. (2022). Briefly, the TDP-43 RNA aptamer conjugated to ATTO 595 (sequence: CGGUGUUGCU_ATTO590) was designed using an *in silico* approach that performs large-scale predictions of protein-RNA interactions based on physico-chemical properties; the algorithm is called *cat*RAPID (Cirillo et al., 2016). Aptamer was synthesised and purified via high performance liquid chromatography by ATDBio Ltd. (Zacco et al., 2022). Spinal cord sections were incubated for at least 1 h with the fluorescently conjugated aptamer (1 μM) after the primary and secondary antibody incubation steps and just prior to imaging.

### Airyscan confocal microscopy

**Figure 1:**
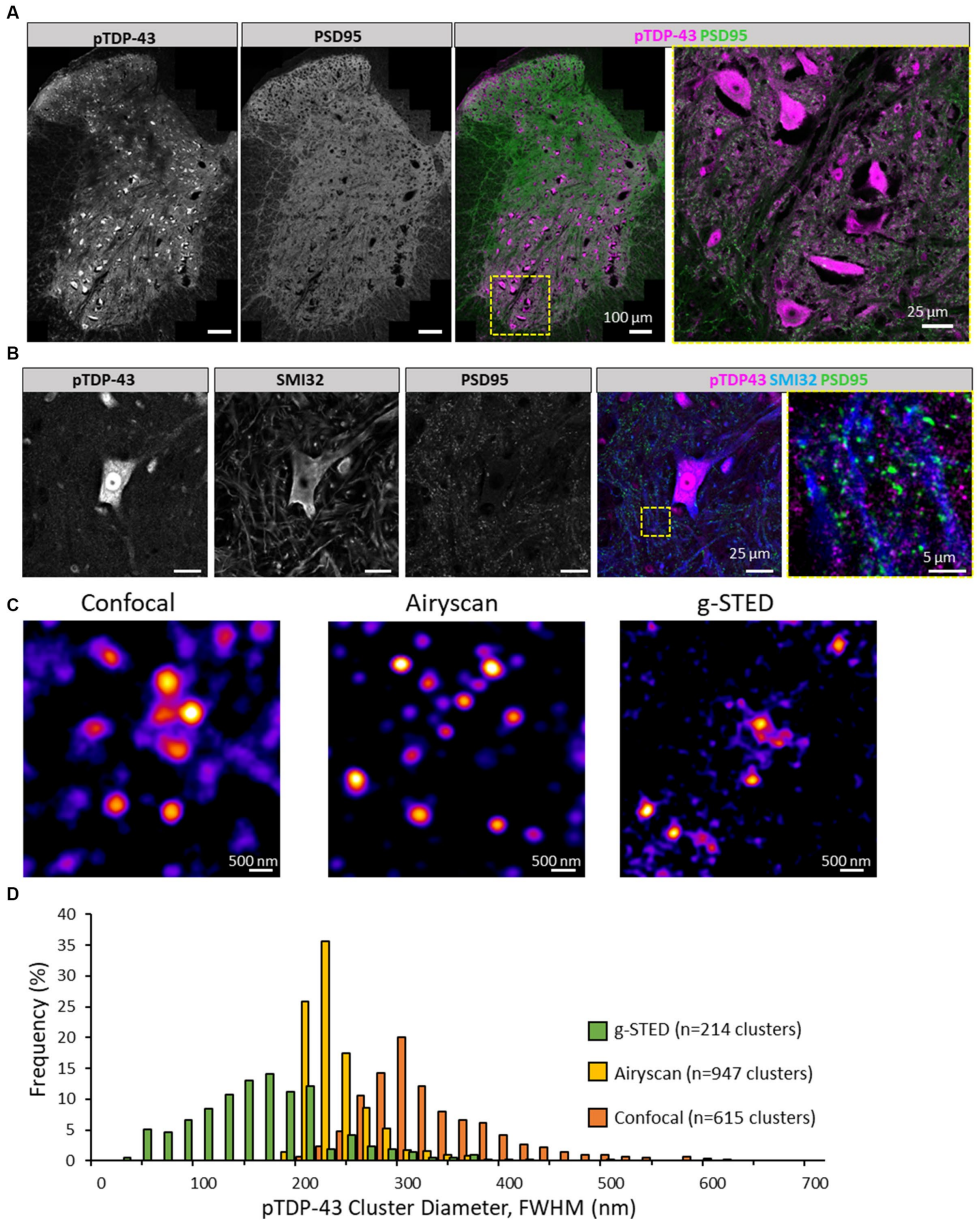
Visualising pTDP-43 clusters in the mouse spinal cord. **(A)** Immunolabelling of pTDP-43 in the lumbar spinal cord of mice expressing PSD95-eGFP. Image was captured using high-resolution tiling of multiple images across a hemisection of the mouse spinal cord. In the ventral horn (yellow square) large pTDP-43 positive cells observed, showing some nuclear and somatic labelling. There is also considerable pTDP-43 expression outside cell bodies near the surrounding green fluorescence from the synaptic marker, PSD95. **(B)** Motor neurons in the ventral horn, labelled with SMI32, are shown to display pTDP-43 expression. Outside the soma of the motor neuron (yellow box) pTDP-43 expression is observed in small clusters nearby SMI32-positive processes and their associated synapses as shown through the PSD95 marker. **(C)** pTDP-43 clusters in the neuropil of the mouse spinal cord were visualised using laser scanning Confocal microscopy (left), Airyscan (centre) and g-STED (right). **(D)** Histogram of the average diameter of pTDP-43 clusters as detected using the three different microscopy techniques.

**Figure 2:**
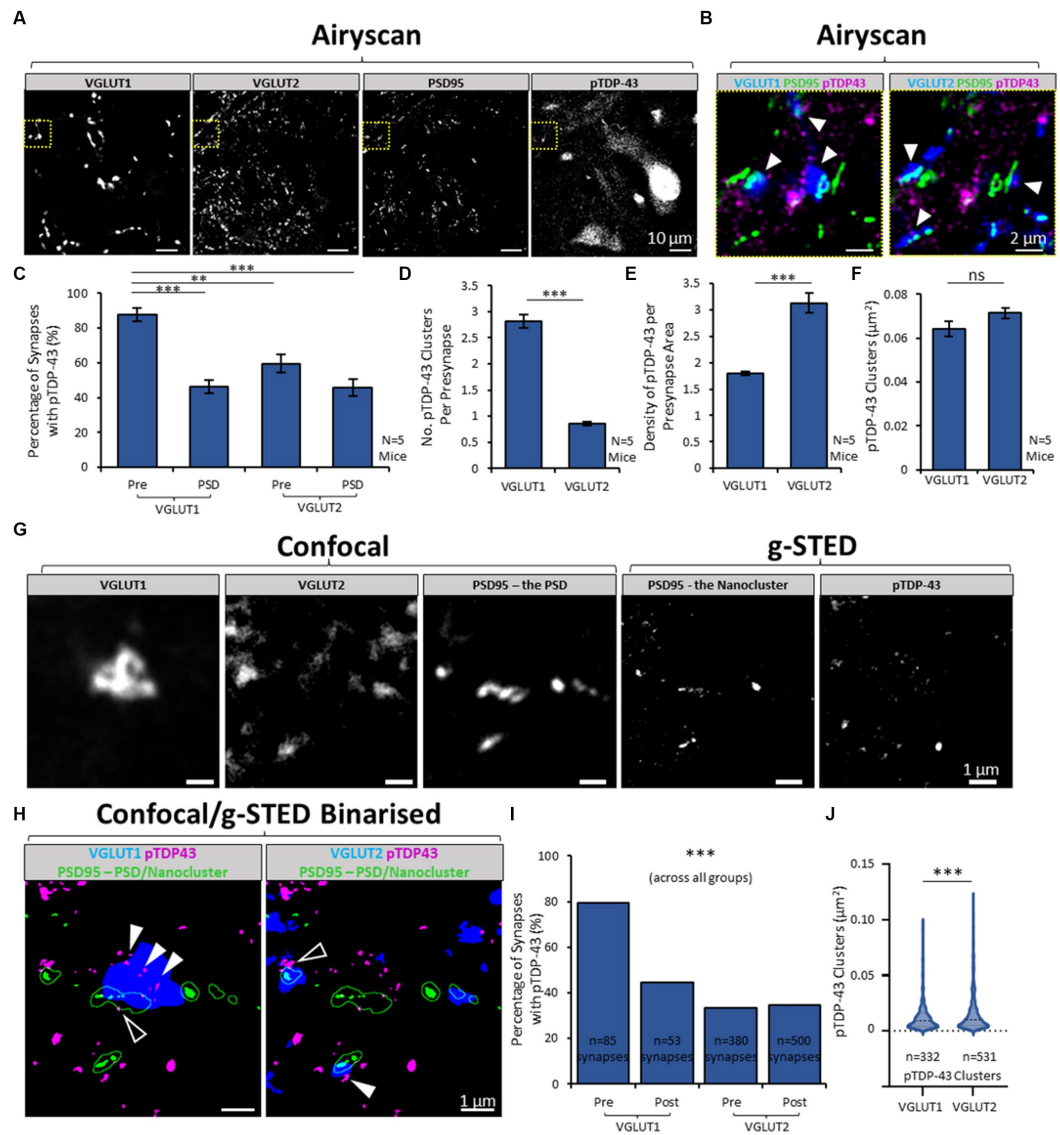
Heterogeneity in presence of pTDP-43 between synapse subtypes in the mouse spinal cord. **(A)** Airyscan acquisition were obtained of VGLUT1, VGLUT2, PSD95 and pTDP-43 in the mouse spinal cord, to visualise and then quantify the expression of pTDP-43 clusters inside two different populations of excitatory synapses. **(B)** Merged-channel images are shown of the yellow cropped boxes in panel **A**, first showing VGLUT1, PSD95 and pTDP-43 (left) and then showing VGLUT2, PSD95 and pTDP-43 (right). VGLUT1 and VGLUT2 label two distinct populations of synapses in the ventral horn of the mouse spinal cord. Clusters of pTDP-43 (white arrows) can be seen associated with both VGLUT1 and VGLUT2-associated synapses. **(C)** Graph plotting the percentage of different synapse structures containing any pTDP-43 clusters. A One-Way ANOVA and *post-hoc* Tukey’s testing reveals there is significant differences in the presence of pTDP-43, with presynaptic VGLUT1 structures being more frequently associated with pTDP-43 than postsynaptic sites, or VGLUT2 synapses entirely. **(D)** The number of pTDP-43 clusters per presynaptic terminal was quantified and shows that VGLUT1 presynaptic terminals have a greater number of clusters than VGLUT2 presynaptic terminals, tested using a Two-Sample *T*-Test for significance. **(E)** This graph plots the ‘density’ of pTDP-43 clusters in synapses, by normalising the number of TDP-43 clusters inside synapses to the total area covered by the synaptic markers. This graph reveals that VGLUT2 synapses in fact have a greater density of pTDP-43 clusters, due to the size difference between VGLUT1 and VGLUT2 presynaptic terminals. **(F)** The size of pTDP-43 clusters was measured in VGLUT1 and VGLUT2 synapses using the area of the detected structures from thresholding. **(G)** Laser scanning Confocal microscopy was used to visualise VGLUT1, VGLUT2 and PSD95, whilst correlative g-STED microscopy was used to visualise pTDP-43 and PSD95 again in the same acquisition series, to identify synaptic clusters of pTDP-43 with nanoscale precision. Visualising PSD95 with confocal microscopy enables us to determine the overall boundaries of the ‘PSD,’ whilst g-STED resolves the underlying nanoclusters (NCs) of PSD95 that form the basis of neurotransmitter receptor subdomains inside the synapse. **(H)** The images from panel **G** are binarized and merged in two combinations, showing VGLUT1-associated synapses (left) and VGLUT2-associated synapses (right). The PSD (PSD95 imaged with confocal) is shown as a green outline, whilst the PSD95 NCs are shown as filled green structures. Filled white arrows denote presynaptic clusters of pTDP-43, while white outlined arrows show postsynaptic clusters of TDP-43. **(I)** The percentages of synaptic structures (VGLUT1 presynaptic and postsynaptic, VGLUT2 presynaptic and postsynaptic) containing pTDP-43 clusters are plotted. Chi-squared test for associated was used to reveal a significant difference between synapses and their colocalization with pTDP-43. **(J)** The sizes of the pTDP-43 clusters detected inside presynaptic VGLUT1 and VGLUT2 synapses were compared in a violin plot. Using a Mann–Whitney *U* test, pTDP-43 clusters in VGLUT2 were significantly larger than those in VGLUT1 synapses. Statistical significance is denoted in the figures as either: ns, not significant; **p < 0.01; ***p < 0.001.

Confocal microscopy images were captured using a Zeiss LSM800 laser scanning confocal microscope, based on an Axio ‘Observer 7’ microscope, equipped with a 63× 1.4 NA objective lens, an Airyscan super-resolution module plus two individual GaAsP PMT detectors. Illumination was provided by 405, 488, 561, and 640 nm laser lines. For generating 16-bit maps of the spinal cord with high-resolution (Figure 1A), the tiling function was used, with each pixel set to 198 × 198 nm. Images captured of SMI32-positive motor neurons (Figure 1B) were captured using a small tiling array in the ventral horn of a mouse spinal cord section with a higher-resolution (pixel size of 85 × 85 nm). Sub-confocal resolution images for analysing pTDP-43 clusters and synapses (Figures 1C,D, 2A–F, 4A–I) were acquired using the Airyscan detector module. Pixel size was set to 35 nm, with a resultant image size of 77.16 × 77.16 μm. Bi-directional scanning was performed with pixel scan time of 2.04 μs and 2x averaging. A subset of multi-channel Z-stack images were acquired with a voxel size of 42.6 × 42.6 × 150 nm, with each channel acquired sequentially per Z-plane.

### Gated-stimulated emission depletion microscopy and correlative confocal microscopy

Multi-channel confocal and gated-stimulated emission depletion (g-STED) microscopy was performed using the Leica SP8 SMD g-STED microscope available at the Edinburgh Super-Resolution Imaging Consortium (ESRIC) hosted by Heriot Watt University. Excitation was provided by a CW super-continuum white light laser source. Depletion was provided by a 594 and 775 nm laser. Images were acquired with a 1.4 NA 100× oil STED objective lens. The optical zoom was set to provide a resultant image pixel size was 20 nm. Fluorescence was detected using a Leica Hybrid detector, gated at 0.5–8 ns for g-STED images. Confocal and g-STED images were captured sequentially (confocal then g-STED) from regions of interest.

### Aptamer DNA-PAINT

Single-molecule localisation microscopy (SMLM) was performed using an Inverted Nikon TI2 microscope (Nikon, Japan) with a 1.49 NA 60x TIRF objective (CFI Apochromat TIRF 60XC Oil, Nikon, Japan) and a ‘perfect focus’ system to autocorrect for z-drift during acquisition. Illumination was provided with 488 (Cobolt MLD 488–200 Diode Laser System, Cobalt, Sweden), 561 (Cobolt DPL561-100 DPSS Laser System, Cobalt, Sweden) and 638 nm (Cobolt MLD Series 638–140 Diode Laser System, Cobolt AB, Solna, Sweden) laser lines (typical powers of 50–100 W cm^−2^). Fluorescence collected was separated from laser excitation using a dichroic mirror [Di01-R405/488/561/635 (Semrock, Rochester, NY, United States)], and was passed through appropriate filters [488 nm: BLP01-488R, FF01-520/44 (Semrock, NY, United States), 561 nm: LP02-568-RS, FF01-587/35 (Semrock, Rochester, NY, United States), 638 nm: FF01-692/40–25 (Semrock, Rochester, NY, United States)]. The fluorescence was then passed through a 2.5× beam expander and recorded on an EMCCD camera (Delta Evolve 512, Photometrics, Tucson, AZ, United States) operating in frame transfer mode (EMGain = 11.5 e^−^/ADU and 250 ADU/photon). The microscope was automated using the open-source microscopy platform Micromanager. TIRF images were acquired of PSD95-eGFP, VGLUT2 and VGLUT1 by acquiring 100 frames with a 50 ms exposure time. Super-resolution images of TDP-43 were generated using Aptamer DNA-PAINT (AD-PAINT) (Whiten et al., 2018). Briefly, the high nanomolar affinity of the aptamer to TDP-43 enables individual binding events to be observed and localised with an accuracy of ~20 nm. The imaging was performed by acquiring 4000 frames at 50 ms exposure time.

### Image analysis

Image analysis was conducted in Fiji Is Just ImageJ (FIJI) (Schindelin et al., 2012). As a standardised approach to measure the sizes of pTDP-43 clusters detected from different microscopy methods (see Figures 1C,D), a publicly available plugin (“Full-with-half-maximum of 2D spots,” developed by Christoph Sommer)^1^ was employed. Briefly, the pTDP-43 clusters were detected using a find-maximum function. Four line profiles (vertically, horizontally and two diagonals) were then drawn spanning the central maxima point of each clusters, and the line profiles were fitted to a gaussian distribution. The full width at half maximum intensity (FWHM) was measured for each line, giving a measure of the diameter of the clusters. The average FWHM from across the four lines was computed to give an overall average diameter. Clusters were excluded from analysis if fit precision (R^2^) of any of the four lines was too low, or minimum or maximum sizes of the clusters were out-with the expected ranges.

To quantify the synaptic expression of pTDP-43 from the Airyscan data set, images underwent background subtraction, Gaussian blurring and manual thresholding to segment structures in each image. A small, customised macro was written to process each image (see Supplementary material 1 for all macros), allow the user to threshold each image manually, and then use the Analyse Particles function to quantify the number and sizes of objects. As manual thresholding was required, the user was blinded to the identity of the mouse or experimental condition. Colocalisation between pairs of markers was determined by redirecting the intensity measurements from one binarized image (for example, VGLUT1) to the binarized image of a different marker (e.g., pTDP-43). Any measure of intensity detected above 0 was then deemed as colocalization (Broadhead et al., 2020, 2022). An adapted version of this macro was used to analyse similar data captured using Confocal/g-STED microscopy. While VGLUT1 and VGLUT2 synapses are largely independent presynaptic structures with little colocalization between them in the ventral horn of the mouse spinal cord, some VGLUT1 terminals showed a small degree of VGLUT2 expression. We therefore defined ‘VGLUT1 synapses’ as VGLUT1-positive structures with PSD95 associated partial-colocalisation, regardless of any VGLUT2 expression; whilst ‘VGLUT2 synapses’ were VGLUT2-positive structures with PSD95 associated partial colocalization and no VGLUT1 expression.

In 3D data sets (Supplementary materials 4, 5), images were processed using the 3D Distance Analysis plugin (DiAna) to determine the spatial colocalization between synapses and pTDP-43 clusters in 3D, including quantifying the number of pTDP-43 clusters inside each synaptic structure (Gilles et al., 2017).

From the SMLM data sets, images were assessed for drift prior to analysis and excluded accordingly. TDP-43-aptamer images first underwent a background subtraction (rolling ball radius 12 pixels). Using the GDSC SMLM plugin, the Peak Fit function was used to detect single molecule fluorescence events to a Gaussian fit. Localisations were filtered based on a minimum signal strength threshold of 20, a minimum photon count threshold of 50, and a fitting precision threshold of 40 nm. Clusters of TDP-43 from single molecule localisation events were detected using Density-Based Cluster Analysis (DBSCAN) (minimum localisation threshold of 5, clustering distance of 40 nm), and their association with synaptic structures was quantified from their spatial overlap with manually thresholded VGLUT1 and VGLUT2 terminals.

### Statistics

**Figure 3:**
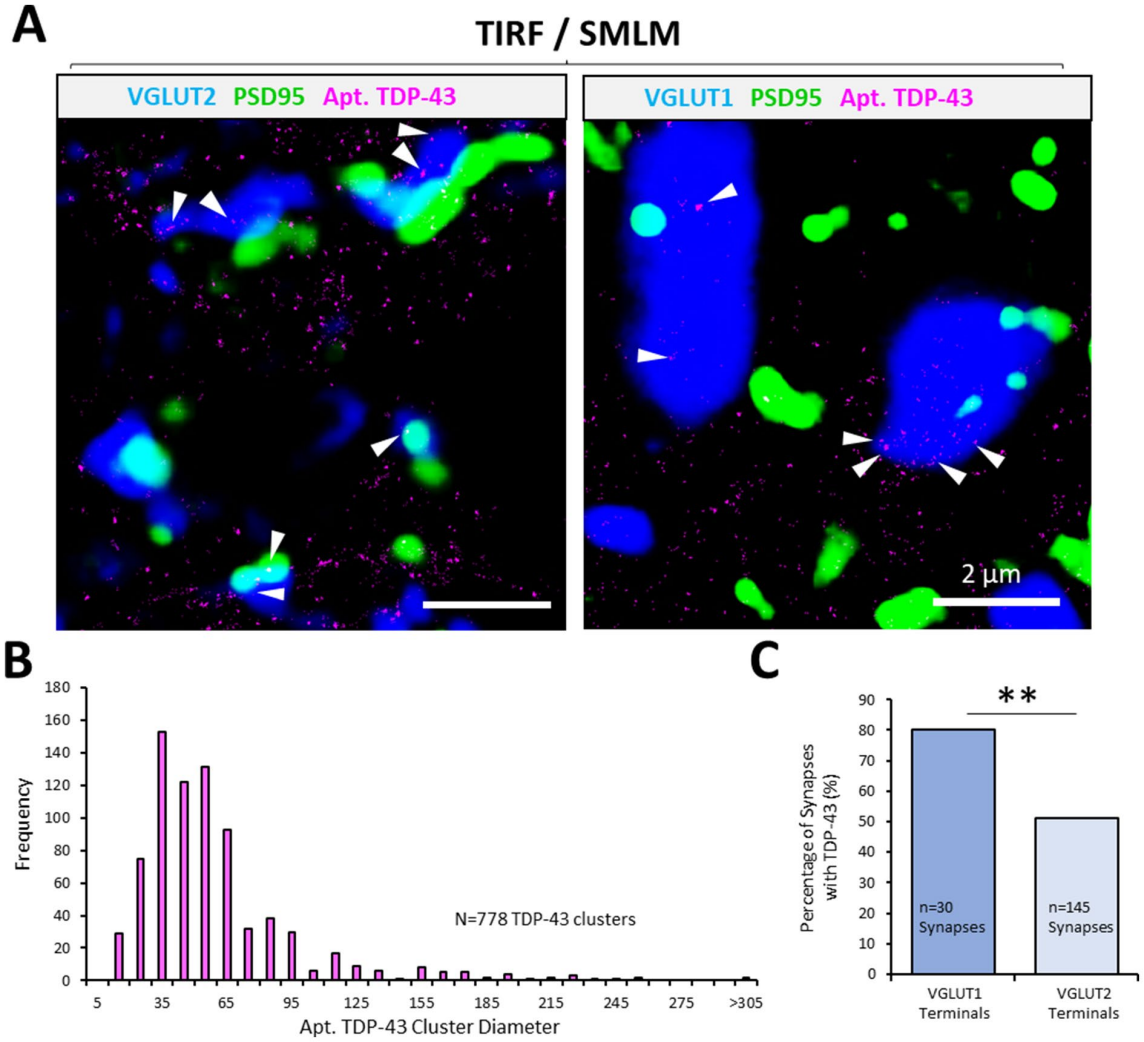
Synaptic pTDP-43 clusters revealed using AD-PAINT. AD-PAINT was used to visualise TDP-43 clysters in PSD95-eGFP mouse spinal cord tissue sections that were either co-immunolabelled with VGLUT1 or VGLUT2 to label different excitatory synapse populations. **(A)** Apt. TDP-43 clusters are rendered from SMLM data, whilst presynaptic and postsynaptic structures are rendered from average projections of the TIRF images captured from their respective channels. pTDP-43 clusters can be identified at both VGLUT1 and VGLUT2 synapse (white arrows). **(B)** Frequency histogram of the sizes of TDP-43 clusters, indicating that the majority of clusters are small 50–60 nm structures. **(C)** The percentage of VGLUT1 and VGLUT2 presynaptic terminals containing TDP-43 cluster is plotted. Chi-squared test for association reveals a statistically significant difference in the presence of the TDP-43 at the two synapse populations. Statistical significance is denoted in the figures as either: ns, not significant; **p < 0.01.

**Figure 4:**
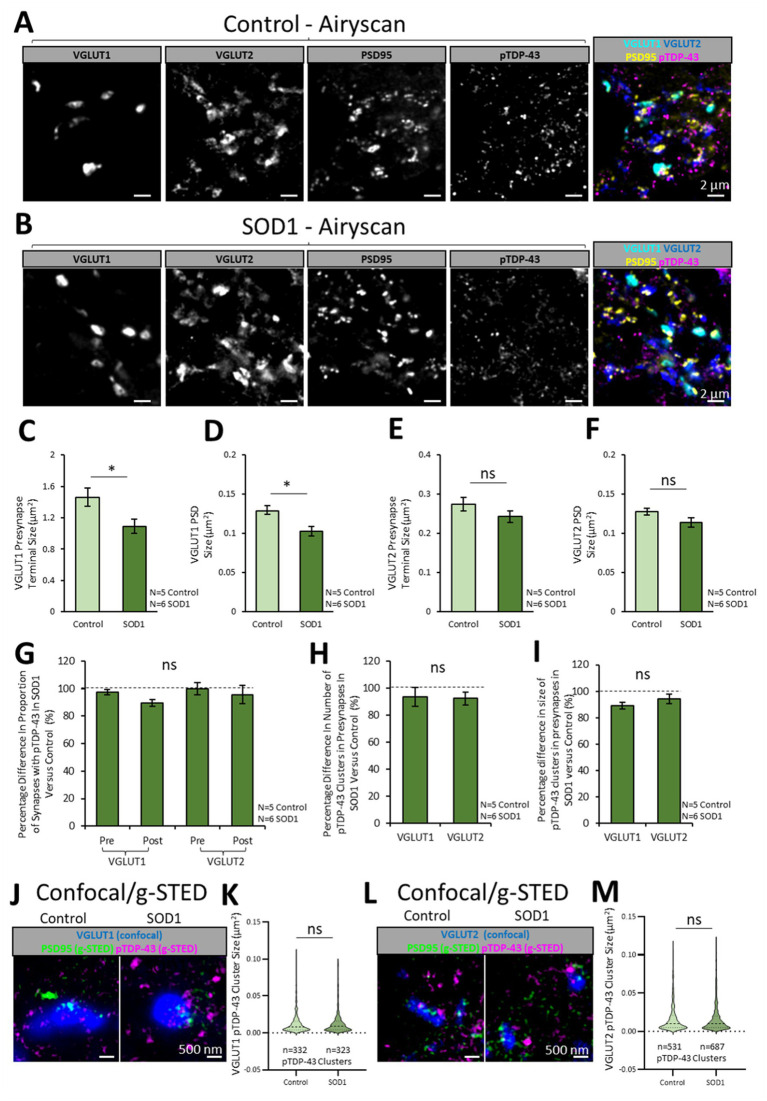
Synaptic pTDP-43 clusters are no different between healthy control and SOD1 mice. To investigate whether the ALS mouse model, SOD1^G93a^ (SOD1), displays any change in the synaptic expression of pTDP-43, excitatory synapses and pTDP-43 clusters were visualised in symptomatic stage SOD1 mice (111  days old) and age matched healthy controls. **(A)** Healthy mouse spinal cord sections expressing PSD95-eGFP were co-labelled for pTDP-43, VGLUT2 and VGLUT1, with a merged imaged on the right showing the excitatory synapse populations and clusters of pTDP-43. **(B)** SOD1 mouse spinal cord sections expressing PSD95-eGFP were similarly labelled to identify pTDP-43 clusters in excitatory synapse populations. **(C)** The graph plots the size of VGLUT1 presynaptic terminals in control and SOD1 mice. A Two-sample T test reveals that VGLUT1 presynaptic terminals are significantly smaller in SOD1 mice compared to healthy controls. **(D)** The graph plots the size of the PSDs associated with VGLUT1 synapses, showing that this population of PSDs is significantly smaller in SOD1 mice compared to healthy controls. **(E)** The graph plots the size of VGLUT2 presynaptic terminals, showing no significant difference in their size between control and SOD1 mice. **(F)** The graph plots the size of PSDs associated with VGLUT2 synapses, showing no significant difference in their size between control and SOD1 mice. **(G)** The graph plots the percentage difference in the proportion synapse structures that contain pTDP-43 clusters in SOD1 mice when normalised to controls (100%). Using a multi-factorial ANOVA statistical test, it is shown there is no difference in the presence of pTDP-43 clusters at VGLUT1 and VGLUT2 presynaptic terminals in SOD1 mice compared to controls. **(H)** The graph plots the percentage difference in the number of pTDP-43 clusters per synapse in SOD1 mice when normalised to controls. The results show there is no difference in the number of pTDP-43 clusters at VGLUT1 and VGLUT2 presynaptic terminals in SOD1 mice compared to controls. **(I)** The graph plots the size of pTDP-43 clusters per synapse in SOD1 mice when normalised to controls. The results show there is no difference in the size of pTDP-43 clusters at VGLUT1 and VGLUT2 presynaptic terminals in SOD1 mice compared to controls. **(J)** VGLUT1-associated excitatory synapses and pTDP-43 clusters were resolved with confocal and g-STED microscopy across a healthy control and SOD1 mouse. **(K)** The violin plot shows the sizes of pTDP-43 clusters associated with VGLUT1 presynaptic terminals in control and SOD1 mice. Using a Mann–Whitney *U* test, it is shown there is no difference in pTDP-43 cluster size between control and SOD1 mice. **(L)** In the same tissue samples, VGLUT2-associated excitatory synapses and pTDP-43 clusters were resolved using confocal and g-STED microscopy in control and SOD1. **(M)** The violin plot shows the sizes of pTDP-43 clusters associated with VGLUT2 presynaptic terminals in control and SOD1 mice. Using a Mann–Whitney *U* test, it is shown there is no difference in pTDP-43 cluster size between control and SOD1 mice. Statistical significance is denoted in the figures as either: ns, not significant; *p < 0.05; **p < 0.01; ***p < 0.001.

Data handling and preparation of graphs was performed in Microsoft Excel. Statistical tests were performed in GraphPad Prism (Version 9.1.0 (21)) or IBM SPSS (Version 28.0.1.1 (15)). Bar charts display error bars denoting the standard error of the mean. Violin plots are used with an inclusive median. Shapiro–Wilk test was used to assess data distribution for normality. A parametric Two-sample *T*-Test was used to compare measures of parameters between two groups (Figures 2D–F, 4C–F); or a non-parametric Mann–Whitney *U* test was used (Figures 2J, 4K,M). Analysis of pTDP-43 presence at four different synaptic structures and subtypes in healthy control mice visualised from Airyscan data sets was performed using a One-Way ANOVA with a post-hoc Tukey’s test (Figure 2C). When analysing these data to compare healthy control with SOD1 mice, a multifactorial Two-way ANOVA was performed (Figures 4G–I). A Chi-squared test for association was used to measure differences in the association of TDP-43 with either VGLUT1 or VGLUT2 synapses from analysis across a pooled number of synapses (Figures 2I, 3C). Comparisons of pTDP-43 cluster size between healthy control and SOD1 mice visualised from the g-STED data set was performed using a Mann–Whitney *U* Test (Figures 4K,M). Data were handled in Microsoft Excel. Where appropriate, the same sizes are denoted in the figures for each data set. Statistical significance is denoted in the figures as either: not significant (ns), *p* < 0.05 (*), *p* < 0.01 (**) or *p* < 0.001 (***). Samples sizes are typically denoted in the figures, either as N = number of animals, or n = number of synapses or clusters.

## Results

### Characterisation of pTDP-43 expression in mouse spinal cord

Immunolabelling of pTDP-43 was performed in upper lumbar spinal cord sections 1–3 (L1-3) obtained from adult (111 days old) control mice that were heterozygous for the PSD95-eGFP knock-in. Two different polyclonal antibodies, raised in rabbit, were tested for labelling pTDP-43 (serine 409/410) in the mouse spinal cord (ProteinTech, 22309–01; and Cosmo Bio, CAC-TIP-PTD-P07). Both antibodies displayed similar labelling patterns in the mouse spinal cord, with some apparent nuclear and somatic labelling of neuronal cells, as well a dense array of puncta in the neuropil that were not present in the negative primary antibody control labelling (Supplementary material 2). The majority of the data reported in this study are from experiments using the Cosmo Bio pTDP-43 antibody, but in some cases additional supportive data were captured using the ProteinTech antibody.

Using high-resolution confocal microscopy, we found that pTDP-43 displays strong labelling of large neurons in the ventral horn of the mouse spinal cord (Figure 1A). pTDP-43 was further shown to display robust labelling in SMI32-positive motor neurons in the ventral horn, not only in the soma and nuclei, but along motor neuron processes and near puncta of the genetically encoded postsynaptic marker, PSD95-eGFP (PSD95) (Figure 1B).

We next characterised the morphology of these pTDP-43 clusters in the neuropil, as assessed using a number of different high-resolution and super-resolution microscopy techniques. These included Zeiss Airyscan, Leica Confocal and g-STED microscopes (Figures 1C,D). The diameter of pTDP-43 clusters was measured using a plugin that detects puncta, generates four lines crossing their centres, fits the intensity of those line profiles to a gaussian function, and calculates the diameter from the FWHM of the fitted profiles (see methods section). pTDP-43 clusters visualised with the confocal microscope displayed a mean diameter of 315 ± 68 nm (*n* = 615 clusters, median: 297 nm; Figures 1C,D). Clusters measured with the Airyscan measured 221 ± 35 nm diameter (*n* = 947 clusters, median: 211 nm; Figures 1C,D). Clusters of pTDP-43 visualised using g-STED microscopy measured 143 ± 65 nm (*n* = 214 clusters, median: 140 nm; Figures 1C,D).

Clusters detected using the alternative pTDP-43 antibody (ProteinTech) appeared very similar in Confocal microscopy compared to those detected using the Cosmo Bio antibody (*U* = 188965, *p* = 0.144), displaying a mean of 318 ± 64 nm (*n* = 586, median: 302 nm, Supplementary materials 3A,B). However, the pTDP-43 clusters detected using the ProteinTech antibody were found to be larger than those detected using the Cosmo Bio antibody when visualised using Airyscan (*U* = 564897, *p* < 0.00001; Supplementary materials 3C,D) or g-STED (*U* = 28389, *p* < 0.00001; Supplementary materials 3E,F).

### Diversity in synaptic expression of pTDP-43

We next determined whether the TDP-43 clusters observed in the neuropil resided inside synapses by visualising pTDP-43 along with synaptic markers using high-resolution and super-resolution microscopy in healthy adult mouse lumbar spinal cords. The genetically encoded PSD95-eGFP marker was used to detect the postsynaptic densities (PSDs) of excitatory synapses (Figures 2A,B). PSD95-eGFP mouse spinal cord sections were immunolabelled with pTDP-43 along with antibodies detecting VGLUT1 and VGLUT2 (Figures 2A,B). Both VGLUT1 and VGLUT2 label distinct populations of presynaptic terminals (also referred to as presynaptic boutons). In the ventral horn of the mammalian lumbar spinal cord, VGLUT1 terminals arise from corticospinal pathways and mechanoreceptive terminals from sensory neurons within the dorsal root ganglia (Todd et al., 2003; Ni et al., 2014). Meanwhile, the majority of VGLUT2 terminals derive from local spinal neurons, with a smaller number corresponding to nociceptive inputs and descending inputs from the rubrospinal and vestibulospinal tracts (Todd et al., 2003; Lagerström et al., 2010; Du Beau et al., 2012). In these data we define synapses as regions where PSD95 ‘PSDs’ and presynaptic terminals (either VGLUT1 or VGLUT2) are colocalised (or partially colocalised).

Using Airyscan microscopy, images of pTDP-43 clusters in excitatory synapses were captured from the ventral horn of lumbar spinal cord sections from healthy control mice (*N* = 5 mice). pTDP-43 clusters were identified in 87.6 ± 3.7% of VGLUT1-positive presynaptic terminals, compared with 59.6 ± 5.0% of VGLUT2-positive presynaptic terminals [*F*(3,16) = 19.9, *p* < 0.0001; Figure 2C]. pTDP-43 were also identified in 46.2 ± 3.7% PSDs associated with either VGLUT1, and 45.7 ± 5.0% of PSDs associated with VGLUT2 terminals. There was a greater number of pTDP-43 clusters per VGLUT1 presynaptic terminal compared to VGLUT2 presynaptic terminals [*T*(8) = 15.2, *p* < 0.0001; Figure 2D]. Conversely, when the number of pTDP-43 clusters inside VGLUT1 or VGLUT2 presynaptic terminals was normalised to the total area covered per image by either VGLUT1 or VGLUT2, it was found that VGLUT2 terminals displayed a significantly greater density of pTDP-43 than VGLUT1 terminals [*T*(8) = 7.1, *p* < 0.001; Figure 2E]. Thus the smaller, more abundant VGLUT2 terminals have a higher density of pTDP-43 clusters than VGLUT1 terminals.

When pTDP-43 cluster size was determined using thresholding to measure individual cluster area, pTDP-43 clusters were not statistically different in their sizes between VGLUT1 and VGLUT2-associated presynaptic terminals, although there was a non-significant trend toward increased cluster size in presynaptic VGLUT2 compartments [*T*(8) = 1.7, *p* = 0.121; Figure 2F].

These findings were replicated in a complementary set of experiments. The pTDP-43 clusters were instead labelled using the ProteinTech antibody, VGLUT1 and VGLUT2 labelling was performed separately in different tissue sections, and the data was collected and analysed using 3D data sets acquired using the Airyscan (Supplementary materials 4A,B). From these data, we similarly observed heterogeneity in the percentage of different synapse structures that contained pTDP-43 clusters [*F*(3,16) = 26.1, *p* < 0.001; Supplementary material 4C] or the number of pTDP-43 clusters per synapse structure [*F*(3,16) = 19.2, *p* < 0.001; Supplementary materials 4D]. Overall, these data show that VGLUT1 presynaptic structures are more commonly associated with pTDP-43 clusters than postsynaptic structures or VGLUT2 synapses.

We also used confocal/g-STED microscopy to visualise the synaptic expression of pTDP-43 clusters in the ventral horn of one mouse spinal cord tissue section with greater resolution. Confocal images were obtained of VGLUT1, VGLUT2 and PSD95-eGFP to visualise excitatory presynaptic terminals and the ‘PSDs’ of excitatory synapses (Figure 2G). g-STED microscopy was used to visualise pTDP-43 clusters and also to resolve the ‘Nanoclusters’ of PSD95-eGFP that comprise the PSD (Figure 2G). The numbers, and sizes, of pTDP-43 clusters were then analysed inside different synapse subtypes and domains (Figure 2H). Using this higher-resolution approach, it was similarly observed that 79% of presynaptic VGLUT1 terminals contained pTDP-43 clusters whilst only 30–45% of PSDs and presynaptic VGLUT2 terminals contained pTDP-43 clusters (Figure 2I). Chi-squared analysis showed there was a significant difference in the association of pTDP-43 clusters between the different synapse subtypes or compartments [χ(8) = 13665.5, *p* < 0.001; Figure 2I]. From analysis of sizes of 332 pTDP-43 clusters associated with VGLUT1 terminals and 531 clusters associated with VGLUT2 terminals, those observed in VGLUT2 terminals were, on average, significantly larger than those in VGLUT1 terminals (*U* = 101474.5, *p* < 0.001; Figure 2J).

These data confirm that pTDP-43 is localised synaptically and that there is heterogeneity in the presence of pTDP-43 clusters between different, distinct subtypes of synapses and across synaptic compartments.

### Diversity in synaptic expression of TDP-43 resolved using aptamer labelling

Aptamers are sequences of DNA that can be specifically designed to bind to peptide sequences of interest. An anti-TDP-43 aptamer has been developed and tested for use in identifying TDP-43 aggregation (Zacco et al., 2022). AD-PAINT was used to investigate whether aptamer-labelled TDP-43 displayed a differential distribution in synapse subtypes. PSD95-eGFP mouse spinal cord tissue sections were immunolabelled with either VGLUT1 or VGLUT2 to visualise excitatory PSDs and different presynaptic terminals, respectively. The tissue was then incubated in the anti-TDP-43 aptamer (Apt. TDP-43), and images were captured (across 2 sections from the same mouse). Single molecule localisations of Apt. TDP-43 were overlayed with averaged projections of the fluorescence signals of the synaptic markers visualised in TIRF (Figure 3A). The resolution of this approach tested across 3 representative images was calculated as 37.8 ± 0.08 nm, as measured by Fourier Ring Correlation (Nieuwenhuizen et al., 2013). Using this approach, Apt. TDP-43 clusters were observed within synaptic compartments, notably inside presynaptic terminals (Figure 3A). Using DBSCAN cluster analysis to detect clusters of Apt. TDP-43 associated with either VGLUT1 or VGLUT2 synapses, the median diameter across 778 clusters was measured as 50.5 nm, and ranged from 14 to 333 nm in diameter (Figure 3B). From analysis of 30 VGLUT1 synapses and 145 VGLUT2 synapses, we observed that VGLUT1 terminals were more significantly associated with Apt. TDP-43 clusters (80%) compared to VGLUT2 terminals (51%) (χ(1) = 8.5, *p* = 0.004; Figure 3C). These data further demonstrate that TDP-43 is expressed and localised in clusters at synapses, with a prominent presence in VGLUT1 presynaptic terminals.

### Synaptic expression of pTDP-43 in a mouse model of ALS

Various reports indicate TDP-43 pathology may play a role in the synaptic dysfunction in ALS. The SOD1 ALS mouse model displays many cellular and behavioural phenotypes that recapitulate the human disease, including synaptic dysfunction, motor neuron loss and progressive motor control deficits. Although many reports indicate that the SOD1 mouse model displays no TDP-43-associated pathology, the synaptic presence of TDP-43 in this model has not been investigated using nanoscopy techniques that may detect more subtle changes.

We therefore assessed the subsynaptic expression of pTDP-43 in populations of excitatory synapses in the lamina IX motor pools of upper lumbar spinal cords from both healthy control (*N* = 5) and SOD1 mice (*N* = 6). The SOD1 mice were cross-bred with PSD95-eGFP mice such that all progeny were heterozygous for the PSD95-eGFP mutation, with some of the progeny expressing the SOD1 mutation. The control littermates not expressing the SOD1 mutation were the ones used in the previous sections (Figures 1–3). Mice were aged 16 weeks old (111-days); a time-point we have previously demonstrated to display significant synapse loss (Broadhead et al., 2022). Spinal cords were immunolabelled for pTDP-43, VGLUT2 and VGLUT1 and images were acquired using the Zeiss Airyscan (Figures 4A,B).

VGLUT1-associated presynaptic terminals were significantly smaller in the SOD1 spinal cord tissue compared to control [*T*(9) = 2.9, *p* = 0.017; Figure 4C]. Correspondingly, the PSD95-eGFP PSD’s associated with VGLUT1 presynaptic terminals were significantly smaller in SOD1 mice compared to controls [*T*(9) = 2.4, *p* = 0.041; Figure 4D]. While the VGLUT2 presynaptic terminals and PSDs were also slightly reduced in size in SOD1 mice, these differences were not statistically significant [VGLUT2 terminal size: *T*(9) = 1.3, *p* = 0.238; Figure 4E; PSD size: *T*(9) = 1.7, *p* = 0.123; Figure 4F].

A Two-Way ANOVA was used to analyse differences in the association of pTDP-43 clusters with synapses between the two disease conditions (Control versus SOD1) and between different synapse structure types (VGLUT1-associated PSDs and terminals, and VGLUT2-associated PSDs and terminals). The data were plotted as the percentage difference between SOD1 and Control mice (Figure 4G). There was no significant difference in the proportion of synaptic structures that contained pTDP-43 clusters between control and SOD1 mice [*F*(1,39) = 1.0, *p* = 0.319; Figure 4G]. Similarly, there was no significant difference in the number or size of pTDP-43 clusters associated with VGLUT1 and VGLUT2 presynaptic terminals in control and SOD1 mice [number: *F*(1,19) = 1.0, *p* = 0.322, Figure 4H; size: *F*(1,19) = 0.2, *p* = 0.686, Figure 4I]. g-STED microscopy also detected no differences in pTDP-43 cluster sizes between control and SOD1 in VGLUT1 synapses (*U* = 52,480, *p* = 0.638; Figures 4J,K) nor VGLUT2 synapses (*U* = 176,092, *p* = 0.300; Figures 4L,M). Finally, from the complementary 3D Airyscan study undertaken using the alternative pTDP-43 antibody (ProteinTech), using *N* = 5 SOD1 samples for the VGLUT1 labelling and *N* = 6 SOD1 samples for the VGLUT2 labelling, we also observed no significant differences in percentages of synapses with pTDP-43 [*F*(1,37) = 3.0, *p* = 0.092; Supplementary material 5C] or the number of pTDP-43 clusters at different synapses [*F*(1,37) = 1.0, *p* = 0.325; Supplementary material 5D] between control and SOD1 mice. Similarly, the size of pTDP-43 clusters was no different between genotypes.

## Discussion

### Nanoscale synaptic clustering of TDP-43

Our study reveals nanoscale synaptic expression of TDP-43 in the ventral horn of the lumbar spinal cord in adult mice. This was apparent using two different anti-pTDP-43 antibodies and an anti-TDP-43 aptamer, visualised with a range of high-resolution and super-resolution microscopy techniques. While there is considerable literature on the role of TDP-43 in synaptic function in health and disease (Godena et al., 2011; Fogarty et al., 2016; Jiang et al., 2019; Dyer et al., 2021; Bak et al., 2022), our findings offer a subsynaptic characterisation that supports the conclusion that TDP-43 may have direct implications on synaptic function. The synaptic clusters of TDP-43 appear to be diffraction limited in size – measuring 50–60 nm in diameter using AD-PAINT, 100–200 nm in diameter using g-STED microscopy, or 200–250 nm using Airyscan. These measurements are largely consistent with other quantitative assessments of TDP-43 clusters using similar super-resolution microscopy techniques and electron microscopy in mammalian cell lines and tissue (Johnson et al., 2009; Kao et al., 2015; Wong et al., 2022; Zacco et al., 2022). The apparent size of TDP-43 clusters measured is highly dependent on the resolution of the approach. Furthermore, aptamer labels are much smaller than primary-secondary antibody complexes. Therefore, the spatial localisation of the aptamer-conjugated fluorophore to the protein target of interest is more accurate, while antibody approaches may displace the signal from the target protein, resulting in seemingly larger structures. Within each data set captured using a particular method, there was homogeneity in the distribution of TDP-43 cluster sizes, suggesting that synaptic TDP-43 clusters in the mouse spinal cord are relatively conserved in size and shape. Whilst we did observe differences in the sizes of pTDP-43 clusters detected between the two different commercially available antibodies, there was overall consistency in their apparent labelling in the mouse spinal cord tissue. More importantly, following quantitative analysis, both antibodies revealed the same consistent findings of synaptic heterogeneity in pTDP-43 clusters, and confirmed the lack of changes in SOD1 mice compared to healthy controls.

Each method incurs its own advantages and limitations. While AD-PAINT provides optimal resolution of TDP-43 clusters, image acquisition is slow and large data sets are generated that require considerable computational power (>1Gb per acquisition), making processing and analysis complex and time-consuming. Both the Airyscan and g-STED methods offered reasonable improvements in resolution beyond diffraction limited confocal microscopy (though inferior to AD-PAINT) and are relatively ‘user friendly’ for non-specialist microscopists in terms of sample preparation, speed of acquisition, data-set size and analysis.

Nanoscale clusters of TDP-43 were found in both VGLUT1- and VGLUT2-associated presynaptic terminals in regions where motor neurons reside. While TDP-43 appeared to be expressed more predominantly in presynaptic terminals, our data did indicate the presence of postsynaptic TDP-43 clusters, as evidenced by its partial colocalization with PSD95, which has also been observed in other studies (Henstridge et al., 2018). The more frequent presence of TDP-43 clusters in VGLUT1-associated synapses may suggest a more prominent role for TDP-43 in mRNA transport or protein translation at these subtypes of synapses, which are likely to be primary mechanoreceptive terminals or corticospinal afferents (Brown and Fyffe, 1978; Todd et al., 2003; Ni et al., 2014; Schultz et al., 2017).

Despite VGLUT1- synapses being more commonly associated with a greater number of pTDP-43 clusters, such clusters were slightly larger in VGLUT2-associated synapses, when measured using g-STED microscopy. Recent findings using super-resolution microscopy discerned that larger inclusions of TDP-43, not smaller ones, were more strongly associated with neuronal toxicity (Cascella et al., 2022), further demonstrating the utility of super-resolution techniques for capturing nanoscale changes that may precede or predict the formation of larger TDP-43 aggregates and inclusions. The differential expression of TDP-43 between the two subtypes of synapses could incur selective vulnerability of certain synapse subtypes to TDP-43 related neuropathologies, as is observed in ALS. Most prudently, the mechanistic value of a single cluster of pTDP-43 remains to be determined, i.e., what can one single cluster endow the synapse in terms of molecular composition, structure or functionality, compared to those synapses which do not contain pTDP-43 clusters, or indeed those that house multiple clusters?

Our data do not reveal any significant difference in the cluster size or synaptic expression of pTDP-43 in SOD1 mice compared to healthy littermate controls. Previous reports have provided conflicting results regarding the presence or timescale of TDP-43 pathology in the SOD1^G93a^ mouse model. Shan et al. (2009) demonstrated cytoplasmic inclusions of TDP-43 in ‘end-stage’ mice that were approximately 170 days old probed with an anti-TDP-43 antibody. Jeon et al. (2019), demonstrated increased immunoreactivity for pTDP-43 and the C-terminal fragment of TDP-43, but not the N-terminal of TDP-43, in SOD1 mice aged 120 days – the same age used in our study. However, other studies have demonstrated no such TDP-43 pathology at similar early symptomatic stage mice. Similarly, ALS patients carrying SOD1 mutations display a notable absence of TDP-43 pathology (Robertson et al., 2007; Turner et al., 2008; Henstridge et al., 2018). It could be that TDP-43 pathology and synaptopathy in SOD1 mice occur through independent mechanisms, despite the fact that TDP-43 is present at synapses and that findings from human post-mortem studies suggest a correlation between TDP-43 pathology and synaptic loss (Henstridge et al., 2018).

Similar to previous observations by ourselves and others, we observed that the PSDs of excitatory synapses are reduced in size in the SOD1 mice. Interestingly this effect was most prominent in the VGLUT1-associated synapses – with a corresponding reduction in the sizes of the VGLUT1 presynaptic terminals. This finding is mirrored by the heterogeneity in TDP-43 between different synapses, whereby it is more frequently associated with VGLUT1 synapses compared to VGLUT2 synapses. Other synapse subtypes received by motor neurons are also subject to changes, including inhibitory glycinergic synapses (Allodi et al., 2021) and modulatory cholinergic synapses (Herron and Miles, 2012; Witts et al., 2014; Bak et al., 2022). The approaches used in this study would be highly suitable for identifying nanoscopic TDP-43-associated synaptopathy in other such synapse subtypes in a range of ALS models to better understand the underlying mechanisms of selective synaptic vulnerability.

Mutations in a number of different genes, including C9ORF72, TDP43, FUS and SOD1, can all lead to some form of motor neuron disorder or the related cognitive disorder frontotemporal dementia. It is possible that the synapse may act as a key subcellular locus of convergence for these multiple different disease pathways. Recent proteomics studies from human ALS patients identified over 30 different ALS-associated proteins in biochemically extracted synaptosomes (Laszlo et al., 2022). For example, C9orf72 has been shown to interact with the presynaptic protein, Synapsin-I, and perturbed interactions between C9orf72 and Synapsin-I in ALS may be associated with excitatory synapse dysfunction and loss (Devlin et al., 2015; Perkins et al., 2021; Bauer et al., 2022). Super-resolution microscopy techniques have been used to identify the presynaptic localisation of FUS at excitatory synapses, and mutations in FUS are similarly implicated in excitatory synapse dysfunction and loss (Schoen et al., 2015; Deshpande et al., 2019; Sahadevan et al., 2021). There is extensive literature on synaptic dysfunction associated with mutations in the SOD1 gene (Avossa et al., 2006; Naumenko et al., 2011; Herron and Miles, 2012; Fogarty et al., 2015; Fogarty, 2019; Bączyk et al., 2020; Rei et al., 2020; Allodi et al., 2021; Broadhead et al., 2022). Finally, structural and functional synaptic changes have been demonstrated in animal models with TDP-43 mutations (Heyburn and Moussa, 2016; Jiang et al., 2019; Dyer et al., 2021; Bak et al., 2022) and human iPSC-derived neurons from patients with TDP-43 mutations (Devlin et al., 2015), while our findings, along with other published results, support synaptic roles for TDP-43 based on its presynaptic and postsynaptic expression (Johnson et al., 2009; Kao et al., 2015; Wong et al., 2022; Zacco et al., 2022). There is also evidence of pairwise interactions between SOD1 and TDP-43 (Trist et al., 2022), and that misfolding of TDP-43 and FUS proteins may induce pathological misfolding of SOD1 through prion-like mechanisms (Pokrishevsky et al., 2016). The small confined domain of the synapse may increase the chance of protein interactions inducing misfolding, as well as facilitate the prion-like spread of proteins between neurons using exosome-dependent mechanisms (Shimonaka et al., 2016; Westergard et al., 2016), thus further supporting the notion that the synapse could be a highly vulnerable common pathway for different ALS/FTD disease mechanisms. There could be considerable value in generating a more comprehensive anatomical map of different synaptic subtypes, with their expression of different ALS linked proteins fully quantitated with nanoscopic techniques.

In summary, our quantitative super-resolution imaging provides novel characterisation of the subcellular distribution of TDP-43 and nanoscale organisation of protein clusters. These techniques are valuable for understanding the role of TDP-43 in neuronal function. The sensitivity and resolution of such techniques will be valuable in identifying subtle early-stage aggregations of TDP-43, as it is known that complex aggregates form during early pathogenesis of ALS (Ling et al., 2013; Ling, 2018; Chen and Cohen, 2019). Such information will help advance our understanding of early-stage biomarkers of disease and reveal novel therapeutic avenues that target synaptopathy.

## Data availability statement

The raw data supporting the conclusions of this article will be made available by the authors, without undue reservation.

## Ethics statement

The animal study was reviewed and approved by the University of St Andrews Animal Welfare and Ethics Committee.

## Author contributions

MB conceived the hypothesis, wrote the grants to support the research, performed and directed IHC experiments, imaging experiments and analysis, and wrote the manuscript. AA-H, KD and LM performed IHC experiments and high-resolution microscopy and analysis. OK performed IHC experiments, TDP-43 aptamer experiments, and SMLM experiments FZ created PSD-95-eGFP mouse model. SG created PSD-95-eGFP mouse model and feedback on manuscript. MH wrote grants to support the research, performed TDP-43 aptamer experiments and SMLM experiments, assisted with analysis and manuscript writing. GM wrote grants to support the research and assisted with manuscript writing. All authors contributed to the article and approved the submitted version.

## Funding

This work was supported by Motor Neurone Disease (MND) Association UK (Miles/Apr18/863-791), Chief Scientist Office, RS Macdonald Charitable Trust, ALS CURE Project, the European Research Council (ERC) under the European Union’s Horizon 2020 Research and Innovation Programme (695568 SYNNOVATE), Simons Foundation Autism Research Initiative (529085), and the Wellcome Trust (Technology Development grant 202932).

## Conflict of interest

The authors declare that the research was conducted in the absence of any commercial or financial relationships that could be construed as a potential conflict of interest.

## Publisher’s note

All claims expressed in this article are solely those of the authors and do not necessarily represent those of their affiliated organizations, or those of the publisher, the editors and the reviewers. Any product that may be evaluated in this article, or claim that may be made by its manufacturer, is not guaranteed or endorsed by the publisher.

## Footnotes

^1^ https://github.com/sommerc/spots_fwhm
