# Supplementary material for: Synaptic expression of TAR-DNA-binding protein 43 in the mouse spinal cord determined using super-resolution microscopy
Source: Front Mol Neurosci. 2023 Aug 21;16:1027898. doi: 10.3389/fnmol.2023.1027898 (PMC10475998; doi:10.3389/fnmol.2023.1027898)
Supplement: Supplementary file 1 [file Data_Sheet_1.PDF]

Title: Synaptic Expression of TAR-DNA-Binding Protein 43 in the Mouse Spinal Cord Determined Using Super-Resolution Microscopy

Authors:

Matthew J. Broadhead[1,2,3*], Ani Ayvazian-Hancock[1,2] Katherine Doucet[1,2], Owen Kantelberg[4], Sophia Schwantes[1,2], Lesley Motherwell[1,2], Fei Zhu[5], Seth GN Grant[5,6], Mathew H Horrocks[4,7], Gareth B. Miles[1].

Affiliations:

1. School of Psychology & Neuroscience, University of St Andrews, St Andrews, UK.
2. Centre of Biophotonics, University of St Andrews, North Haugh, St Andrews, UK.
3. Edinburgh Super-Resolution Imaging Consortium, Heriot Watt University, Edinburgh, UK.
4. EaStCHEM School of Chemistry, University of Edinburgh, David Brewster Road, Edinburgh EH9 3FJ, UK
5. Genes to Cognition Program, Centre for Clinical Brain Sciences, University of Edinburgh, Edinburgh EH16 4SB, UK.
6. Simons Initiative for the Developing Brain (SIDB), Centre for Discovery Brain Sciences, University of Edinburgh, Edinburgh EH8 9XD, UK.
7. IRR Chemistry Hub, Institute for Regeneration and Repair, University of Edinburgh, Edinburgh EH16 4 UU, UK.

* First and Corresponding Author.

Supplementary Materials.

1. Supplementary Material 1. Analysis Macros
2. Supplementary Material 2. Validation of Immunolabelling of pTDP-43 in the lumbar spinal cord from PSD95-eGFP mice.
3. Supplementary Material 3. Comparison of clusters detected using two different anti-pTDP-43 antibodies.
4. Supplementary Material 4. Complementary analysis of pTDP-43 clusters in excitatory synapses in the ventral horn of the mouse lumbar spinal cord, using the ProteinTechanti-pTDP-43 antibody
5. Supplementary Material 5. Complementary study into pTDP-43 clusters in synapse subtypes in control and SOD1 mice. 3D Z

Supplementary Material 1.

Marco 1. Analysis of pTDP-43 clusters in VGLUT1 and VGLUT2 synapses (2D Airyscan Data):

```
rename("Image");
run("Split Channels");
selectWindow("C1-Image");
rename("VG1");
selectWindow("C2-Image");
rename("TDP");
selectWindow("C3-Image");
rename("PSD");
selectWindow("C4-Image");
rename("VG2");
/// Run BS and Blur
selectWindow("PSD");
run("Duplicate...", " ");
run("Subtract Background...", "rolling=20");
run("Gaussian Blur...", "sigma=1");
selectWindow("VG2");
run("Duplicate...", " ");
run("Subtract Background...", "rolling=30");
run("Gaussian Blur...", "sigma=2");
selectWindow("TDP");
run("Duplicate...", " ");
run("Subtract Background...", "rolling=12");
run("Gaussian Blur...", "sigma=1");
selectWindow("VG1");
run("Duplicate...", " ");
run("Subtract Background...", "rolling=60");
run("Gaussian Blur...", "sigma=2");
```

```
selectWindow("PSD-1");
///PSD Thresholding
setAutoThreshold("Otsu dark");
setOption("BlackBackground", false);
run("Convert to Mask");
run("Watershed");
run("Set Measurements...", "display redirect=None decimal=3");
run("Analyze Particles...", "size=12-Infinity pixel show=Masks exclude");
selectWindow("TDP-1");
///TDP Thresholding
setAutoThreshold("Moments dark");
run("Convert to Mask");
run("Watershed");
run("Set Measurements...", "display redirect=None decimal=3");
run("Analyze Particles...", "size=6-300 pixel show=Masks exclude");
selectWindow("VG2-1");
///VG2 Thresholding
setAutoThreshold("Otsu dark");
run("Convert to Mask");
run("Watershed");
run("Set Measurements...", "display redirect=None decimal=3");
run("Analyze Particles...", "size=30-infinity pixel show=Masks exclude");
selectWindow("VG1-1");
///VG1 Thresholding
setAutoThreshold("Otsu dark");
run("Convert to Mask");
run("Watershed");
run("Set Measurements...", "display redirect=None decimal=3");
run("Analyze Particles...", "size=200-infinity pixel show=Masks exclude");
/// Analyse Stuff
selectWindow("Mask of PSD-1");
```

```
run("Set Measurements...", "area mean display redirect=[Mask of VG2-1] decimal=3");
run("Analyze Particles...", "size=12-Infinity pixel show=Nothing display exclude");
IJ.renameResults("PSD_VG2");
selectWindow("Mask of PSD-1");
run("Set Measurements...", "area mean display redirect=[Mask of TDP-1] decimal=3");
run("Analyze Particles...", "size=12-Infinity pixel show=Nothing display exclude");
IJ.renameResults("PSD_TDP");
selectWindow("Mask of PSD-1");
run("Set Measurements...", "area mean display redirect=[Mask of VG1-1] decimal=3");
run("Analyze Particles...", "size=12-Infinity pixel show=Nothing display exclude");
IJ.renameResults("PSD_VG1");
///VGLUT2 Bit
selectWindow("Mask of VG2-1");
run("Set Measurements...", "area mean display redirect=[Mask of PSD-1] decimal=3");
run("Analyze Particles...", "size=30-Infinity pixel display exclude");
IJ.renameResults("VG2-PSD");
selectWindow("Mask of VG2-1");
run("Set Measurements...", "area mean display redirect=[Mask of TDP-1] decimal=3");
run("Analyze Particles...", "size=30-Infinity pixel show=Nothing display exclude");
IJ.renameResults("VG2_TDP");
///VGLUT1 Bit
selectWindow("Mask of VG1-1");
run("Set Measurements...", "area mean display redirect=[Mask of PSD-1] decimal=3");
run("Analyze Particles...", "size=200-Infinity pixel show=Nothing display exclude");
IJ.renameResults("VG1_PSD");
selectWindow("Mask of VG1-1");
run("Set Measurements...", "area mean display redirect=[Mask of TDP-1] decimal=3");
run("Analyze Particles...", "size=200-Infinity pixel show=Nothing display exclude");
IJ.renameResults("VG1_TDP");
///TDP43 Bit - analyse particles of TDP-43, re-direct to VG1 and VG2 Separately
selectWindow("Mask of TDP-1");
```

```
run("Set Measurements...", "area mean display redirect=[Mask of VG1-1] decimal=3");
run("Analyze Particles...", "size=6-300 pixel show=Nothing display exclude");
IJ.renameResults("TDP_VG1");
selectWindow("Mask of TDP-1");
run("Set Measurements...", "area mean display redirect=[Mask of VG2-1] decimal=3");
run("Analyze Particles...", "size=6-300 pixel show=Nothing display exclude");
IJ.renameResults("TDP_VG2");
```

Marco 2. Analysis of pTDP-43 clusters in VGLUT1 and VGLUT2 synapses (2D Airyscan Data):

```
rename("Image");
run("Split Channels");
selectWindow("C1-Image");
rename("VG1");
selectWindow("C2-Image");
rename("VG2");
selectWindow("C3-Image");
close()
selectWindow("C4-Image");
rename("TDP");
selectWindow("C5-Image");
rename("PSD");
selectWindow("C6-Image");
rename("NC");
/// Run BS and Blur
selectWindow("PSD");
run("Duplicate...", " ");
run("Subtract Background...", "rolling=25");
run("Gaussian Blur...", "sigma=2");
selectWindow("VG2");
run("Duplicate...", " ");
run("Subtract Background...", "rolling=35");
run("Gaussian Blur...", "sigma=2");
selectWindow("TDP");
run("Duplicate...", " ");
run("Subtract Background...", "rolling=10");
run("Gaussian Blur...", "sigma=2");
selectWindow("VG1");
run("Duplicate...", " ");
run("Subtract Background...", "rolling=60");
```

```
run("Gaussian Blur...", "sigma=2");
selectWindow("NC");
run("Duplicate...", " ");
run("Subtract Background...", "rolling=16");
run("Gaussian Blur...", "sigma=2");
selectWindow("PSD-1");
setAutoThreshold("Otsu dark");
///PSD Thresholding – Adjust Manually If Needed
setOption("BlackBackground", false);
run("Convert to Mask");
run("Set Measurements...", "display redirect=None decimal=3");
run("Analyze Particles...", "size=25-Infinity pixel show=Masks exclude");
selectWindow("NC-1");
setAutoThreshold("Moments dark");
///NC Thresholding – Adjust Manually If Needed
setOption("BlackBackground", false);
run("Convert to Mask");
run("Set Measurements...", "display redirect=None decimal=3");
run("Analyze Particles...", "size=12-Infinity pixel show=Masks exclude");
selectWindow("TDP-1");
///TDP Thresholding – Adjust Manually If Needed
setAutoThreshold("Moments dark");
run("Convert to Mask");
run("Watershed");
run("Set Measurements...", "display redirect=None decimal=3");
run("Analyze Particles...", "size=6-300 pixel show=Masks exclude");
selectWindow("VG2-1");
///VG2 Thresholding – Adjust Manually If Needed
setAutoThreshold("Otsu dark");
run("Convert to Mask");
run("Watershed");
```

```
run("Set Measurements...", "display redirect=None decimal=3");
run("Analyze Particles...", "size=40-infinity pixel show=Masks exclude");
selectWindow("VG1-1");
///VG1 Thresholding – Adjust Manually If Needed
setAutoThreshold("Otsu dark");
run("Convert to Mask");
run("Watershed");
run("Set Measurements...", "display redirect=None decimal=3");
run("Analyze Particles...", "size=300-infinity pixel show=Masks exclude");
/// Analyse Stuff
selectWindow("Mask of PSD-1");
run("Set Measurements...", "area mean display redirect=[Mask of VG2-1] decimal=3");
run("Analyze Particles...", "size=25-Infinity pixel show=Nothing display exclude");
IJ.renameResults("PSD_VG2");
selectWindow("Mask of PSD-1");
run("Set Measurements...", "area mean display redirect=[Mask of TDP-1] decimal=3");
run("Analyze Particles...", "size=25-Infinity pixel show=Nothing display exclude");
IJ.renameResults("PSD_TDP");
selectWindow("Mask of PSD-1");
run("Set Measurements...", "area mean display redirect=[Mask of VG1-1] decimal=3");
run("Analyze Particles...", "size=25-Infinity pixel show=Nothing display exclude");
IJ.renameResults("PSD_VG1");
///VGLUT2 Bit
selectWindow("Mask of VG2-1");
run("Set Measurements...", "area mean display redirect=[Mask of PSD-1] decimal=3");
run("Analyze Particles...", "size=40-Infinity pixel display exclude");
IJ.renameResults("VG2-PSD");
selectWindow("Mask of VG2-1");
run("Set Measurements...", "area mean display redirect=[Mask of TDP-1] decimal=3");
run("Analyze Particles...", "size=40-Infinity pixel show=Nothing display exclude");
IJ.renameResults("VG2_TDP");
```

```
///VGLUT1 Bit
selectWindow("Mask of VG1-1");
run("Set Measurements...", "area mean display redirect=[Mask of PSD-1] decimal=3");
run("Analyze Particles...", "size=300-Infinity pixel show=Nothing display exclude");
IJ.renameResults("VG1_PSD");
selectWindow("Mask of VG1-1");
run("Set Measurements...", "area mean display redirect=[Mask of TDP-1] decimal=3");
run("Analyze Particles...", "size=300-Infinity pixel show=Nothing display exclude");
IJ.renameResults("VG1_TDP");
///TDP43 Bit - analyse particles of TDP-43, re-direct to VG1 and VG2 Separately
selectWindow("Mask of TDP-1");
run("Set Measurements...", "area mean display redirect=[Mask of VG1-1] decimal=3");
run("Analyze Particles...", "size=6-300 pixel show=Nothing display exclude");
IJ.renameResults("TDP_VG1");
selectWindow("Mask of TDP-1");
run("Set Measurements...", "area mean display redirect=[Mask of VG2-1] decimal=3");
run("Analyze Particles...", "size=6-300 pixel show=Nothing display exclude");
IJ.renameResults("TDP_VG2");
//// Distance Based Stuff
///STED Analysis - PSD Bit
selectWindow("Mask of PSD-1");
run("Set Measurements...", "area mean display redirect=PSD decimal=3");
run("Analyze Particles...", "size=30-Infinity pixel exclude add");
run("Set Measurements...", "area mean display redirect=PSD decimal=3");
id = getImageID();
setAutoThreshold("Default");
for (i=0 ; i<roiManager("count");
i++) {
   selectImage(id);
roiManager("select", i);
run("Analyze Particles...", "size=30-Infinity pixel show=Nothing display");
```

```
}
IJ.renameResults("PSD-PSD");
/// STED Analysis - NC Bit
selectWindow("Mask of NC-1");
run("Set Measurements...", "area mean shape integrated display redirect=NC decimal=3");
id = getImageID();
setAutoThreshold("Default");
for (i=0 ; i<roiManager("count");
i++) {
   selectImage(id);
roiManager("select", i);
run("Analyze Particles...", "size=8-Infinity pixel show=Nothing display");
}
IJ.renameResults("PSD-NC");
```

Macro 3. Analysis of pTDP-43 colocalisation with VGLUT1 Synapses (3D):

```
rename("Image");
run("Split Channels");
selectWindow("C3-Image");
rename("PSD");
run("Duplicate...", "duplicate");
selectWindow("C2-Image");
rename("TDP");
run("Duplicate...", "duplicate");
selectWindow("C1-Image");
rename("VG1");
run("Duplicate...", "duplicate");
selectWindow("VG1-1");
run("Subtract Background...", "rolling=60 stack");
run("Gaussian Blur 3D...", "x=2 y=2 z=2");
selectWindow("TDP-1");
run("Subtract Background...", "rolling=12 stack");
run("Gaussian Blur 3D...", "x=1 y=1 z=1");
selectWindow("PSD-1");
run("Subtract Background...", "rolling=20 stack");
run("Gaussian Blur 3D...", "x=1 y=1 z=1");
///PSD Thresholding – Adjust Manually If Needed
setAutoThreshold("Moments dark");
setOption("BlackBackground", false);
run("Convert to Mask", "method=Moments background=Dark");
selectWindow("TDP-1");
///TDP Thresholding – Adjust Manually If Needed
setAutoThreshold("Moments dark");
run("Convert to Mask", "method=Moments background=Dark");
run("Watershed", "stack");
selectWindow("VG1-1");
```

///VG1 Thresholding – Adjust Manually If Needed

setAutoThreshold("Moments dark");

run("Convert to Mask", "method=Moments background=Dark");

///

Diana = Segment PSD Labelled A = 50-200000

Valide, Segment, exclude XY

Diana = Segment TDP Labelled B = 20 - 2000

Valide, Segment, exclude XY

Diana = Segment VG1 Labelled B = 500 - 900000

Valide, Segment, exclude XY

Macro 4. Analysis of pTDP-43 colocalisation with VGLUT2 Synapses (3D):

```
rename("Image");
run("Split Channels");
selectWindow("C3-Image");
rename("PSD");
run("Duplicate...", "duplicate");
selectWindow("C2-Image");
rename("TDP");
run("Duplicate...", "duplicate");
selectWindow("C1-Image");
rename("VG2");
run("Duplicate...", "duplicate");
selectWindow("VG2-1");
run("Subtract Background...", "rolling=30 stack");
run("Gaussian Blur 3D...", "x=2 y=2 z=2");
selectWindow("TDP-1");
run("Subtract Background...", "rolling=12 stack");
run("Gaussian Blur 3D...", "x=1 y=1 z=1");
selectWindow("PSD-1");
run("Subtract Background...", "rolling=20 stack");
run("Gaussian Blur 3D...", "x=1 y=1 z=1");
///PSD Thresholding – Adjust Manually If Needed
setAutoThreshold("Moments dark");
setOption("BlackBackground", false);
run("Convert to Mask", "method=Moments background=Dark");
selectWindow("TDP-1");
///TDP Thresholding – Adjust Manually If Needed
setAutoThreshold("Moments dark");
run("Convert to Mask", "method=Moments background=Dark");
run("Watershed", "stack");
selectWindow("VG2-1");
```

///VG1 Thresholding – Adjust Manually If Needed

setAutoThreshold("Moments dark");

run("Convert to Mask", "method=Moments background=Dark");

Diana = Segment PSD Labelled A = 50-200000

Valide, Segment, exclude XY

Diana = Segment TDP Labelled B = 20 - 2000

Valide, Segment, exclude XY

Diana = Segment VG2 Labelled B = 200 - 900000

Valide, Segment, exclude XY

Supplementary Material 2.

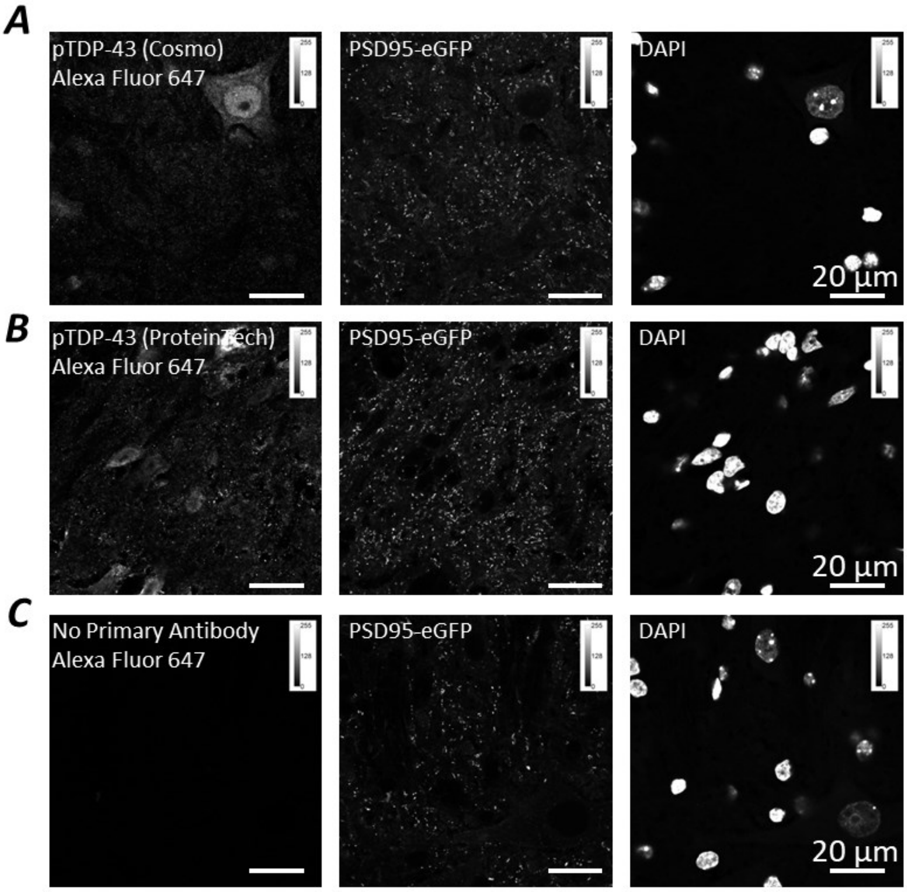

Validation of immunolabelling of pTDP-43 in the lumbar spinal cord from PSD95-eGFP – expressing mice.

A. Immunolabelling of pTDP-43 (Cosmo Bio antibody), alongside the PSD95-eGFP expression and DAPI labelling. B. Immunolabelling of pTDP-43 (ProteinTech antibody), alongside PSD95-eGFP and DAPI labelling. C. Control labelling in which the primary antibody was omitted, but the secondary antibody (Alexa Fluor 647) still included. The intensity of the DAPI and PSD95 images was set at the same scale for all the images with respect to each channel. The display intensity of each channel (DAPI, PSD95 or the pTDP43 labelling was normalized for each channel for comparison of relative expression.

Supplementary Material 3.

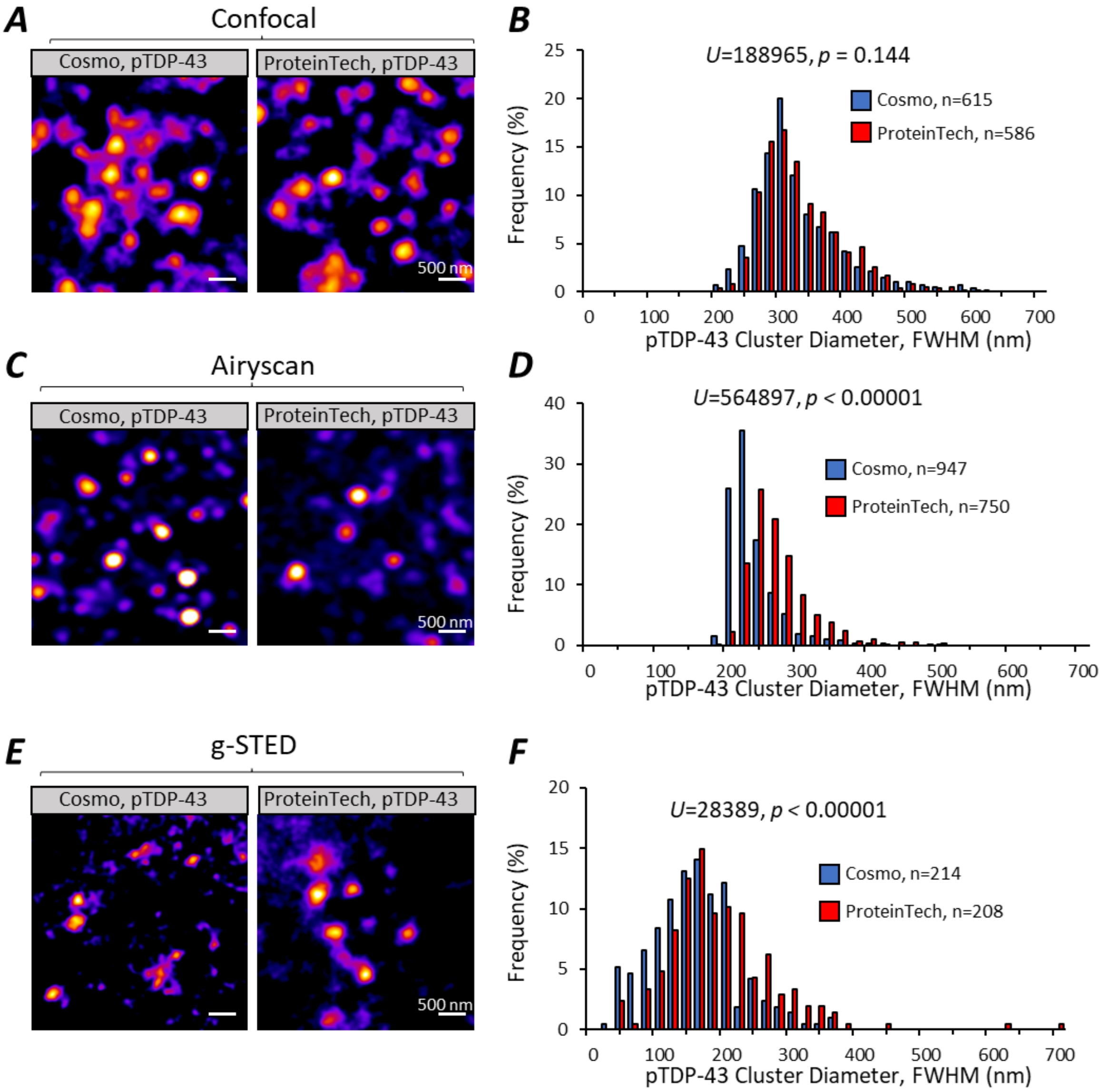

Comparison of clusters detected using two different anti-pTDP-43 antibodies.
A. pTDP-43 clusters visualised using confocal microscopy, labelled with either the Cosmo antibody (left) or the ProteinTech antibody (right). **B.** Frequency histogram shows there is no significant difference in the sizes of the pTDP-43 clusters between the two different antibodies, detected using confocal microscopy. **C.** pTDP-43 clusters detected using the two antibodies, visualised using the Airyscan. **D.** Frequency histogram shows that pTDP-43 clusters are significantly larger when detected using the ProteinTech antibody, compared to those detected using the Cosmo antibody. **E.** pTDP-43 clusters detected using the two different antibodies, visualised with g-STED microscopy. **F.** Frequency histogram shows that pTDP-43 clusters detected using the ProteinTech antibody are significantly larger when visualised using g-STED microscopy.

Supplementary Material 4.

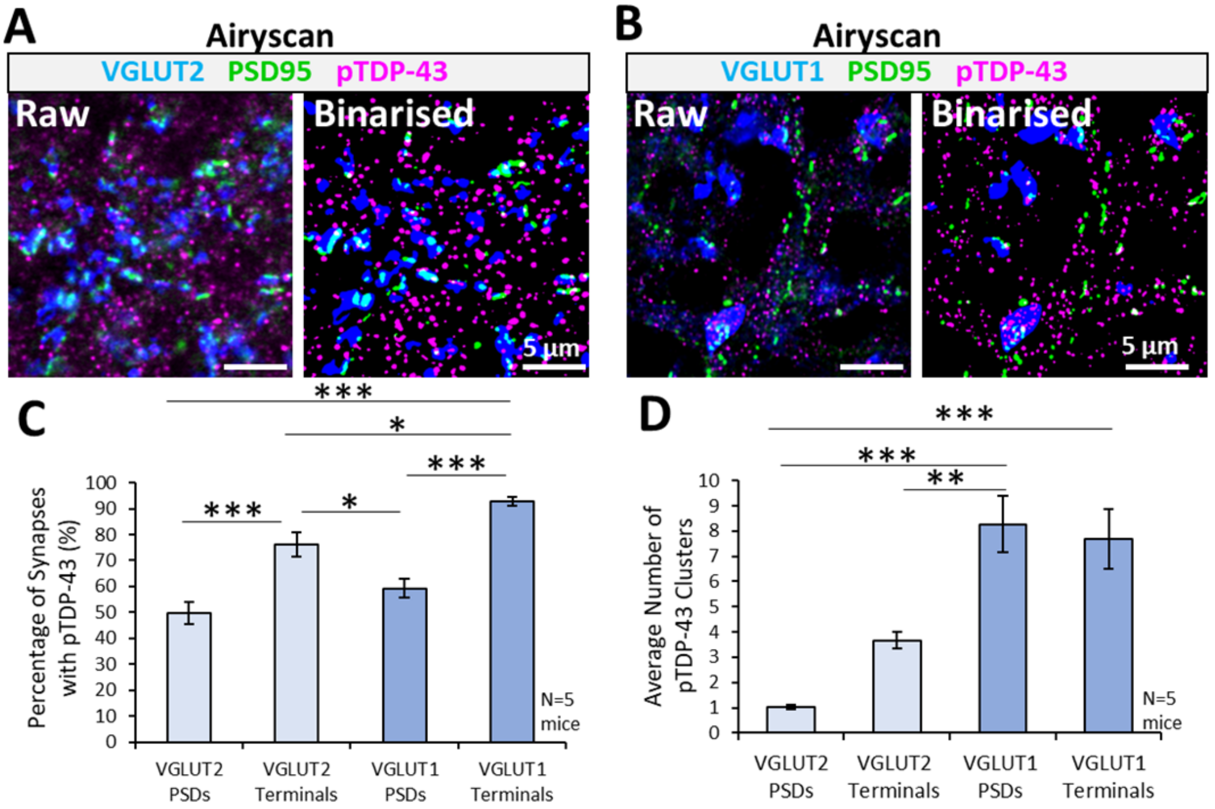

Complementary analysis of pTDP-43 clusters in excitatory synapses in the ventral horn of the mouse lumbar spinal cord, using the ProteinTech anti-pTDP-43 antibody.

A. Raw and binarized images of VGLUT2, PSD95 and pTDP-43 collected using Airyscan. **B**. Raw and binarized images of VGLUT1, PSD95 and pTDP-43 collected using Airyscan. **C**. The percentages of different excitatory synapse structures containing pTDP-43 are plotted. There is a significant difference in the pTDP-43 colocalization with different synapse structures ($F(3,16)=26.1$, $p<0.001$). **D**. The average number of pTDP-43 clusters per synapse structure is plotted. There is a significant difference in the number of pTDP-43 clusters between different synapse structures ($F(3, 16)=19.2$, $p<0.001$).

Supplementary Material 5

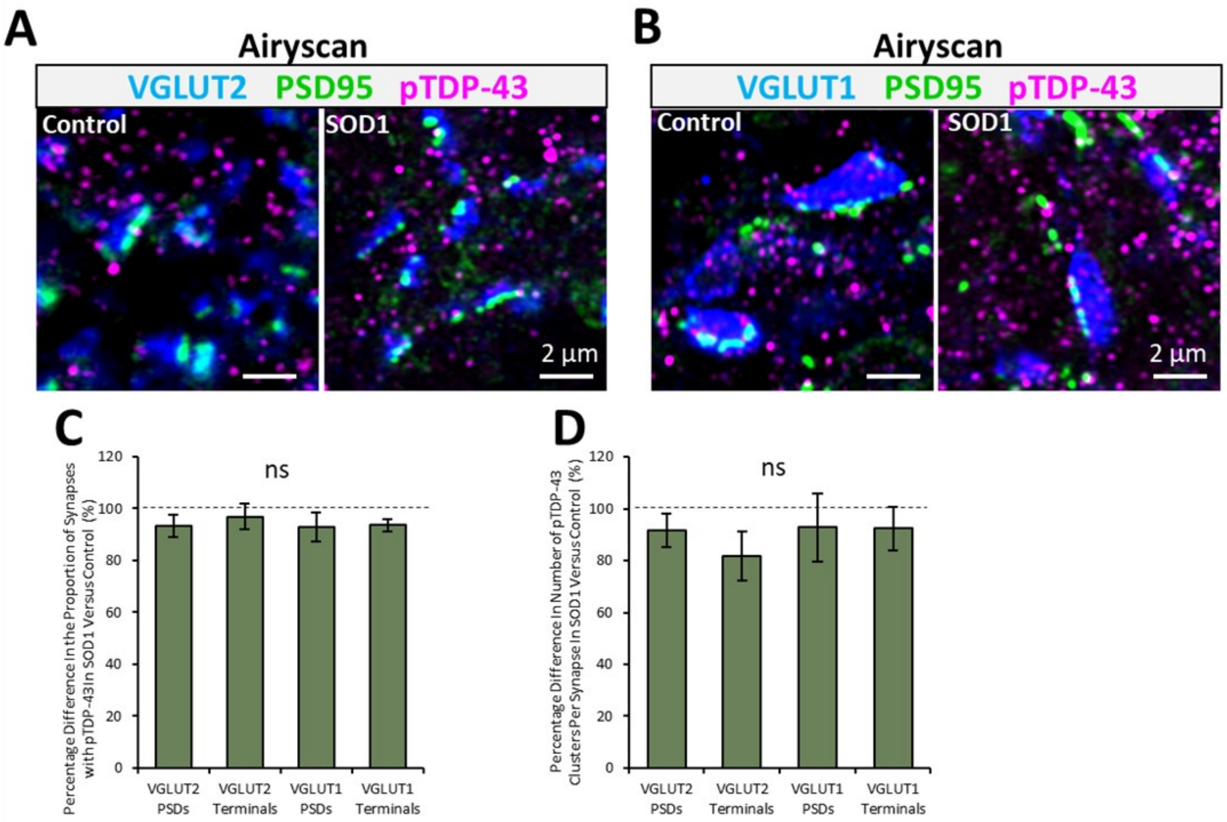

Complementary study into pTDP-43 clusters in synapse subtypes in control and SOD1 mice.

3D Z-stack acquisitions were obtained using airyscan microscopy to quantify the presence of pTDP-43 in different synapse subtypes and structures. **A**. Airyscan images of VGLUT2, PSD95 and pTDP-43 in Control (left) and SOD1 (right) mouse tissue sections. Images were collected from N=5 control and N=6 SOD1 mice. **B**. Airyscan images of VGLUT1, PSD95 and pTDP-43 in Control (left) and SOD1 (right) mouse tissue sections. Images were collected from N=5 control and N=5 SOD1 mice. **C**. The percentage of synapse structures containing pTDP-43 clusters in SOD1 mice is plotted as a percentage difference compared to controls (100%). There is no significance difference in the presence of pTDP-43 at synapses in control and SOD1 mice. **D**. The number of pTDP-43 clusters in different synapse structures in SOD1 mice is plotted as a percentage difference compared to controls (100%). There is no significance difference in the presence of pTDP-43 at synapses in control and SOD1 mice.
